# Hip Fracture Surgery: Does Type of Anesthesia Matter?

**DOI:** 10.1155/2013/252356

**Published:** 2013-06-02

**Authors:** Rizwan Haroon Rashid, Adil Aijaz Shah, Amarah Shakoor, Shahryar Noordin

**Affiliations:** ^1^Section of Orthopedics, Department of Surgery, Aga Khan University Hospital, Karachi 74800, Pakistan; ^2^Aga Khan Medical College, Aga Khan University Hospital, Karachi 74800, Pakistan

## Abstract

*Background*. Hip fracture surgery is a common procedure, and the geriatric population with its multiple comorbid conditions is at most at risk of developing anesthesia-related complications. Data on the impact of type anesthesia on postoperative morbidity and mortality is limited. The effects of regional and general anesthesia on postoperative outcomes need to be clearly elucidated. *Methods*. In this study, all patients who underwent dynamic hip screw (DHS) fixation for intertrochanteric fractures, between January 2005 and December 2010, at the Aga Khan University Hospital, were included. Patients were divided into two groups; group A included those patients who received general anesthesia, and group B consisted of patients who had received regional anesthesia. The two groups were compared for differences in morbidity, mortality, and intraoperative complications based on the type of anesthesia administered. *Results*. During this period, 194 patients underwent DHS fixation. One hundred and seven patients received general anesthesia whereas eighty-seven patients received regional anesthesia. The mean operative time was significantly lower in the group receiving regional anesthesia (1.25 ± 0.39 hrs) as compared to those who received general anesthesia (1.54 ± 0.6 hrs) (*P* < 0.05). There were no statistically significant differences in the rates of wound infections, length of hospital stay, postoperative ambulation status, intraoperative blood loss, postoperative complications, and mortality between the regional and general anesthesia groups. *Conclusion*. Even though administration of regional anesthesia was positively correlated with shorter operative duration, the type of anesthesia was not found to affect surgical outcomes in the two study groups. Based on these results, we recommend that anesthesia should be tailored to individual patient requirements.

## 1. Introduction

It is common for a regular orthopedic surgical facility to operate on several patients with a fractured hip on a weekly basis. Although few of these are traumatic fractures in relatively young patients, however, a vast majority of patients presenting for fixation of such fractures are considerably senescent. The frequency of hip fractures is seeing an increasing trend in many countries across the globe partly due to an increase in the average life expectancy. This means that the percentage of elderly populace at risk for falls and subsequent hip fractures is also on a multifold rise [1, 2]. Furthermore, for every male with a fractured hip there are four women presenting with the same ailment on account of osteoporosis [3]. 

As hip fracture surgery is primarily undertaken on elderly patients, most of the patients undergoing the procedure are likely to have multiple comorbidities in the form of hypertension, diabetes, respiratory, metabolic or cardiac diseases. Hence, hip fracture surgeries are often high risk procedures whether internal fixation is undertaken or the femoral head is being replaced via an arthroplasty. It has been reported that such patients are at high risk of morbidity, mortality and postsurgical disability. The statistics report grim figures of approximately 5% patients dying during hospitalization and 10% dying within 30 days due to appalling pulmonary and cardiovascular complications [4–7]. Therefore one should aggressively take preemptive measures to lowering the risks for these patients. The choice of anesthesia therefore becomes an important variable that can be potentially optimized for each patient. Anesthesia used is either general anesthesia; with the airway maintained by a face mask, laryngeal mask airway (LMA), or endotracheal tube (ET) tube after induction and ventilation being spontaneous or mechanical; or regional where a spinal injection of a local anesthetic or an epidural is used.

A multitude of studies have been done in the past, debunking various myths associated with general versus regional anesthesia particularly when routine orthopedic procedures such as hip fracture surgeries are considered. However, most studies have failed to prove one form of anesthesia as being superior to the other based on the outcomes [8, 9]. We hypothesized that regional anesthesia may improve outcomes due to avoidance of intubation and mechanical ventilation, significantly reduce blood loss and improve postoperative analgesia. Contrariwise, general anesthesia was assumed to be attributed with a much better hemodynamic stability when compared with regional anesthesia. We planned this study to further analyze whether outcomes such as rates of wound infection, length of hospital stay, post- operative ambulation status, intra operative blood loss, postoperative complications, and mortality indeed had an association with the type of anesthesia used.

## 2. Methodology

We reviewed medical records of all patients with intertrochanteric femur fractures admitted to our University Hospital from January 2005 to December 2010 who underwent dynamic hip screw fixation using closed reduction techniques. Patients were divided into two groups based on the type of anesthesia used for each patient. Group A consisted of patients receiving general anesthesia, and Group B included patients receiving regional anesthesia for the same surgical procedure. Both groups had an equal distribution of undisplaced and displaced fractures (data not shown). Outcomes studied included operative time (skin to skin), rates of wound infection, length of hospital stay, postoperative ambulation status, intra operative blood loss, postoperative complications and mortality. SPSS version 19 was used for statistical analysis.

## 3. Results

194 patients underwent hip fracture surgery with dynamic hip screw fixation during this period.  Table 1 shows that the demographics between the two groups were equally distributed and were statistically not significant

The most significant finding of our study was related to operative time in both groups depending on the routes of anesthesia used. For 107 patients in the GA group, the mean operative time was 1.54 hours, while mean operative time for 87 patients in the regional anesthesia group was 1.24 hours (*P* < 0.05) as shown in Table 2. 

There were no statistically significant differences in the rates of wound infections, length of hospital stay, postoperative ambulation status, intraoperative blood loss, postoperative complications and mortality between the regional and general anesthesia groups as shown in Table 3.

## 4. Discussion

The choice of anesthesia for various surgical procedures is an issue of continuing debate. Evidence relating to one type of anesthesia surpassing its the other for a procedure as common as hip fracture surgery remains sketchy and available data is conflicting and inconclusive. The general lack of consensus regarding the superiority of regional over general anesthesia exists mostly as a result of the inherent difficulty that prevails when designing a comparative study for this purpose [10–14]. The escalating rate of fragility fractures of the hip and the associated perioperative morbidity and mortality, especially amongst the elderly, calls for swift and conclusive recommendations to be drawn to address this growing public health problem [15, 16].

Since hip fractures are more common in the elderly, the mode of anesthesia administered during corrective surgery largely depends on the functional status and any preexisting conditions. This makes randomization of study subjects ethically questionable and, hence, difficult. Most studies published on the subject matter have been retrospective data reviews or observational in nature [17]. The advanced age and the nature and severity of comorbidities most elderly subjects suffer from makes it difficult to quantify any benefits regional anesthesia may offer over general anesthesia in the long run. Comorbid conditions also put patients at a higher risk of developing complications [18]. According to evidence presented in the recent literature, regional anesthesia remains the anesthesia of choice for older and ailing patients [19]. Thus, mode of anesthesia is generally tailored according to a patient's requirement at the discretion of the anesthesiologist. Randomization becomes impossible in such situation.

It has been hypothesized by a number of authors that the superiority of regional anesthesia such as spinal, epidural, or neural blockade lies in the lack of need for intubation and mechanical ventilation. Increased respiratory morbidity in patients receiving general anesthesia may be positively correlated with endotracheal intubation and its association with respiratory infections, such as pneumonia, in the long run [19–21]. This has also been proven in a large scale, multicenter study conducted in 126 hospitals in New York. Not only was the incidence of pulmonary complications lower in the regional anesthesia arm of the study but the in-hospital mortality was lower with regional anesthesia compared to general anesthesia (6.8% versus 8.1%) as well [22]. Regional anesthesia also has the added advantage of being able to provide better postoperative analgesia and faster recovery [23].

A systematic review scrutinizing a myriad of variables in the management of hip fracture patients was conducted by Beaupre et al. Amongst other things, this review was able to show that the type of anesthesia administered was associated with risk of mortality postoperatively. Regional anesthesia offered the benefit of low mortality and had minimal side effects such as postoperative delirium which is increasingly seen with elderly patients on whom general anesthesia has been used. Beaupre et al. also noticed a low level of deep venous thromboembolism (DVT), pulmonary embolism, pneumonia and decreased requirements for transfusion intraoperatively in patients receiving regional anesthesia [19]. The reduced incidence of DVT in such patients is a remarkable finding for which many mechanisms have been hypothesized [24]. According to one explanation, reduction in sympathetic tone to the lower limbs allows unimpeded blood flow preventing venous stasis and development of clots [25], whereas other authors have postulated that regional anesthesia directly affects coagulability [26]. However, the routine and judicious use of DVT prophylaxis (early mobilization, compression stockings and low molecular weight heparin) postoperatively contravenes any protective effects regional anesthesia may have to offer [18, 27].

Although the literature on the subject matter seems to be somewhat skewed towards favoring regional anesthesia, especially in the elderly patient clientele, the significance of general anesthesia cannot be overlooked [19]. General anesthesia provides a more stable operative course, and the ensuing hemodynamic stability has been associated with a decreased incidence of cerebrovascular accidents and hypotensive episodes. However, with regional anesthesia fluid administration can tightly controlled according to physiological needs and prevents excessive and often unnecessary hydration of the patient [18]. Other, multicenter studies, however, have debunked this notion altogether due to lack of concrete evidence [28, 29]. Postoperative delirium is an often suggested side effect of general anesthesia, rarely reported in the literature. It is one complication that should be taken in to consideration but should not dictate the choice of anesthesia under consideration [30, 31].

Much like the results of our study, a large scale trial consisting of just over 6000 patients, conducted by O'Hara et al., revealed no differences in outcomes in the two arms of patients, 60 years or older [32]. A major contributory factor to this statistically insignificant difference is the varied group of patients presenting to our center with hip fractures. Due to the diverse range of ages (14–98 years, with a mean age of 65) of the patients undergoing this procedure at our center, it seems that age had a role to play since younger individuals have a higher capacity for physiological compensation and, thus, fare better postoperatively as compared to older individuals [33].

A similar trial conducted by Shih et al. incorporated 335 octogenarians and reported a higher morbidity and rate of postoperative complications associated with the administration of general anesthesia. However, the type of anesthesia had no bearing on postoperative mortality [34]. In a study of 18,000 patients, Neuman et al. reported regional anesthesia to produce fewer adverse effects and postoperative complications when compared with data obtained from patients who had undergone surgery with general anesthesia [22]. Another finding of the trial conducted by Shih and colleagues [34], which has been corroborated by our study and has been well established in the published literature [23], is a significantly reduced duration of operative time. This finding is contrary to some studies which found a decrease in operative time with general anesthesia when compared with regional anesthesia [18].

Any study seeking to evaluate the supremacy of one type of anesthesia over another would most likely be inherently flawed. Firstly, lack of a standardized scoring system that considers various patient parameters allows for institutional bias, where one type of anesthesia may be exceedingly preferred over the other. Secondly, the ethical dilemma of randomizing study subjects would take decision making out of the hands of the anesthetist and prevent patients from receiving anesthesia best suited to their needs based on their health status. Thirdly, defining reliable primary and secondary outcomes is difficult. The thirty-day postoperative mortality has served as a useful endpoint, however; advances and improvements in peri-operative care have made differences in mortality rate, rendered as a result of the type of anesthesia employed, unobservable. Secondary outcomes such as duration of hospital stay and return to mobility are likely to be affected by external factors such as need for nursing care, physical therapy, and in-hospital management of co-morbid conditions. The lack of validated and standardized measures of recovery is the need of the hour. Finally, an ethical dilemma that limits the practicality of designing an RCT is the difficulty in recruiting individuals in the study. Since hip fractures are more commonly seen in the elderly, it would be unethical to include patients with cognitive impairments, excluding a significant chunk (25%–45%) of all patients presenting with hip fractures [17]. 

Our study has a number of caveats. Due to the retrospective nature of our study, the dataset obtained may have subtle unobservable differences that would have been difficult to quantify, which may have affected the overall outcome. The small sample size of this study has made it difficult to appreciate significant differences between the two study populations, which would otherwise become appreciable in a larger sample. The complexity of each procedure was not ascertained which may have had some bearing on outcome and duration of surgery as well. Pure anesthesia-related variables including hypoxia, urinary retention, post operative pain, acute confusional state could not be assessed due to the retrospective nature of the study.

This study adds to the plethora of literatures already available. Evidence favoring one type of anesthesia over the other is scarce and often conflicting [8, 9]. Even though regional anesthesia has been found to be marginally superior to general anesthesia in most studies, the issue still remains open to debate. The morbidity as well as mortality associated with hip fractures and its corrective interventions, especially in the elderly population, needs to be addressed emergently. Prospective studies on a larger patient population, employing best practice guidelines and standardized measures of outcomes, are the need of the hour. 

## 5. Conclusion

Regional anesthesia showed a significantly positive association with shorter operative time. However, the type as well as mode of anesthesia was not found to affect surgical outcomes in the two study groups. Based on these results until more data is available, we recommend that anesthesia should be tailored to individual patient requirements to optimize patient outcomes.

## Figures and Tables

**Table 1:** 

	Gender of the patient	
	Male	Female
Type of anesthesia		
GA	56	51
Regional	42	45

**Table 2:** 

	GA	Regional	*P* value
Mean operative time in hours	1.54 ± 0.6	1.24 ± 0.39	<0.01
Mean length of stay (days)	9.35 ± 9.0	8.63 ± 3.6	0.484
Estimated blood loss (mL)	928 ± 360	912 ± 400	0.758

**Table 3:** 

	Postoperative complications					Total
	Wound Infection	UTI	Death	DVT	None	
Type of anesthesia						
GA	2	3	4	0	98	107
Regional	2	5	5	1	74	87

Total	4	8	9	1	172	194

## References

[1] Cooper C, Campion G, Melton LJ (1992). Hip fractures in the elderly: a world-wide projection. *Osteoporosis International*.

[2] Lancaster HO (1990). *Expectations of Life*.

[3] Parker MJ, Pryor GA (1993). *Hip Fracture Management*.

[4] Lawrence VA, Hilsenbeck SG, Noveck H, Poses RM, Carson JL (2002). Medical complications and outcomes after hip fracture repair. *Archives of Internal Medicine*.

[5] Roche JJW, Wenn RT, Sahota O, Moran CG (2005). Effect of comorbidities and postoperative complications on mortality after hip fracture in elderly people: Prospective Observational Cohort Study. *British Medical Journal*.

[6] Parker M, Johansen A (2006). Hip fracture. *British Medical Journal*.

[7] Radcliff TA, Henderson WG, Stoner TJ, Khuri SF, Dohm M, Hutt E (2008). Patient risk factors, operative care, and outcomes among older community-dwelling male veterans with hip fracture. *Journal of Bone and Joint Surgery A*.

[8] Covert CR, Fox GS (1989). Anaesthesia for hip surgery in the elderly. *Canadian Journal of Anaesthesia*.

[9] Sorenson RM, Pace NL (1992). Anesthetic techniques during surgical repair of femoral neck fractures: a meta-analysis. *Anesthesiology*.

[10] Berggren D, Gustafson Y, Eriksson B (1987). Postoperative confusion after anesthesia in elderly patients with femoral neck fractures. *Anesthesia and Analgesia*.

[11] Davis FM, Laurenson VG (1981). Spinal anaesthesia or general anaesthesia for emergency hip surgery in elderly patients. *Anaesthesia and Intensive Care*.

[12] Juelsgaard P, Sand NPR, Felsby S (1998). Perioperative myocardial ischaemia in patients undergoing surgery for fractured hip randomized to incremental spinal, single-dose spinal or general anaesthesia. *European Journal of Anaesthesiology*.

[13] McKenzie PJ, Wishart HY, Dewar KMS (1980). Comparison of the effects of spinal anaesthesia and general anaesthesia on postoperative oxygenation and perioperative mortality. *British Journal of Anaesthesia*.

[14] McLaren AD, Stockwell MC, Reid VT (1978). Anaesthetic techniques for surgical correction of fractured neck of femur. A comparative study of spinal and general anaesthesia in the elderly. *Anaesthesia*.

[15] Braithwaite RS, Col NF, Wong JB (2003). Estimating hip fracture morbidity, mortality and costs. *Journal of the American Geriatrics Society*.

[16] Johnell O, Kanis JA (2004). An estimate of the worldwide prevalence, mortality and disability associated with hip fracture. *Osteoporosis International*.

[17] White SM, Griffiths R, Moppett I (2012). Type of anaesthesia for hip fracture surgery—the problems of trial design. *Anaesthesia*.

[18] Urwin SC, Parker MJ, Griffiths R (2000). General versus regional anaesthesia for hip fracture surgery: a meta-analysis of randomized trials. *British Journal of Anaesthesia*.

[19] Beaupre LA, Jones CA, Saunders LD, Johnston DWC, Buckingham J, Majumdar SR (2005). Best practices for elderly hip fracture patients: a systematic overview of the evidence. *Journal of General Internal Medicine*.

[20] Rodgers A, Walker N, Schug S (2000). Reduction of postoperative mortality and morbidity with epidural or spinal anaesthesia: results from overview of randomised trials. *British Medical Journal*.

[21] Hunter JD (2006). Ventilator associated pneumonia. *Postgraduate Medical Journal*.

[22] Neuman MD, Silber JH, Elkassabany NM, Ludwig JM, Fleisher LA (2012). Comparative effectiveness of regional versus general anesthesia for hip fracture surgery in adults. *Anesthesiology*.

[23] Parker MJ, Handoll HH, Griffiths R (2004). Anaesthesia for hip fracture surgery in adults. *Cochrane Database of Systematic Reviews*.

[24] Thorburn J, Louden JR, Vallance R (1980). Spinal and general anaesthesia in total hip replacement: frequency of deep vein thrombosis. *British Journal of Anaesthesia*.

[25] Shimosato S, Etsten BE (1969). The role of the venous system in cardiocirculatory dynamics during spinal and epidural anesthesia in man.. *Anesthesiology*.

[26] Modig J, Borg T, Bagge L, Saldeen T (1983). Role of extradural and of general anaesthesia in fibrinolysis and coagulation after total hip replacement. *British Journal of Anaesthesia*.

[27] Planes A, Vochelle N, Fagola M, Feret J, Bellaud M (1991). Prevention of deep vein thrombosis after total hip replacement. The effect of low-molecular-weight heparin with spinal and general anaesthesia. *Journal of Bone and Joint Surgery B*.

[28] Bode RH, Lewis KP, Zarich SW (1996). Cardiac outcome after peripheral vascular surgery: comparison of general and regional anesthesia. *Anesthesiology*.

[29] Christopherson R, Beattie C, Frank SM (1993). Perioperative morbidity in patients randomized to epidural or general anesthesia for lower extremity vascular surgery. *Anesthesiology*.

[30] Riis J, Lomholt B, Haxholdt O (1983). Immediate and long-term mental recovery from general versus epidural anesthesia in elderly patients. *Acta Anaesthesiologica Scandinavica*.

[31] Williams-Russo P, Sharrock NE, Mattis S, Szatrowski TP, Charlson ME (1995). Cognitive effects after epidural vs general anesthesia in older adults: a randomized trial. *Journal of the American Medical Association*.

[32] O’Hara DA, Duff A, Berlin JA (2000). The effect of anesthetic technique on postoperative outcomes in hip fracture repair. *Anesthesiology*.

[33] Gilbert TB, Hawkes WG, Hebel JR (2000). Spinal anesthesia versus general anesthesia for hip fracture repair: a longitudinal observation of 741 elderly patients during 2-year follow-up. *American Journal of Orthopedics*.

[34] Shih YJ, Hsieh CH, Kang TW, Peng SY, Fan KT, Wang LM (2010). General versus spinal anesthesia: which is a risk factor for octogenarian hip fracture repair patients?. *International Journal of Gerontology*.

